# Dealing with the Persistent Pathogenic Issues of Dry Eye Disease: The Importance of External and Internal Stimuli and Tissue Responses

**DOI:** 10.3390/jcm12062205

**Published:** 2023-03-13

**Authors:** Maurizio Rolando, Stefano Barabino, Giuseppe Giannaccare, Pasquale Aragona

**Affiliations:** 1 Ocular Surface Centre, ISPRE Oftalmica Istituto di Medicina Oftalmica, 16129 Genoa, Italy; 2 Centro Superficie Oculare e Occhio Secco, Ospedale Sacco, University of Milan, 20133 Milano, Italy; 3 Department of Ophthalmology, University Magna Graecia of Catanzaro, 88100 Catanzaro, Italy; 4 Eye Clinic, Department of Biomedical Science, University of Messina, 98121 Messina, Italy

**Keywords:** dry eye, para-inflammation, inflammation, homeostasis, cytokine

## Abstract

The immune system plays a central role in protecting the ocular surface from exogenous and endogenous insults, maintaining tissue homeostasis thanks to the mechanism of para-inflammation. This physiological adaptive response may induce resident macrophages/monocytes to produce cytokines and growth factors in order to promote epithelial cell recovery. In case of well-controlled para-inflammation, caused by a low amount of stress, cell viability and function are maintained. When stress becomes too intense, there is a response characterized by the activation of autophagic pathways and consequent cell death. Dysregulated homeostasis and chronic sub-clinical inflammation are the starting points for the development of a stable, chronic inflammatory disease, which leads to ocular surface damage, and, in turn, to the onset or progression of chronic dry eye disease (DED). The long-term management of DED should consider all of the pathogenic issues involved in the disease, including the control of persistent external or internal stresses that are capable of activating and maintaining the para-inflammatory adaptive mechanisms, potentially leading to full-blown inflammation. Dysregulated para-inflammation can be corrected by means of the prolonged use of tear substitutes containing minimal doses of safe corticosteroids or other anti-inflammatory molecules (e.g., corticosteroid, cyclosporine) in order to re-equilibrate ocular surface homeostasis.

## 1. Introduction

The ocular surface is the bodily mucosa that is most exposed to external stimuli during the daytime, and the tear film covering it encounters an enormous number of free radicals and the oxidative stress induced by environmental pollution [1]. In particular, its prolonged exposure to evaporation for several hours a day (when the eye is open between two successive blinks) impairs the health of the ocular surface, affecting the ability of the film to counteract environmental oxidative stress. Tear evaporation is the main cause of tear break-up, and the length of tear break-up time (BUT) plays a fundamental role in epithelial well-being and quality of vision [2]. 

Increased tear water evaporation is typical of all forms of dry eye disease (DED) [3], and it leads to a loss of tear fluid and increased tear film osmolarity, both of which are a source of disturbing stimuli and tissue stress for the ocular surface epithelia. Furthermore, as tear clearance is usually reduced in DED, tear film accumulates noxious agents coming from the outside environment or due to internal metabolic activity, so that tears themselves may become toxic to the ocular surface (toxic tears) [4,5].

## 2. Hormesis and Para-Inflammation—An Adaptive Means of Reacting to Stimuli

The immune system plays a central role in protecting the ocular surface from exogenous and endogenous insults by maintaining tissue homeostasis, and any dysfunction or dysregulation may lead to various immune-related diseases. In 2008, Medzhitov discussed the development and characteristics of inflammation, suggesting that there is an intermediate state between basal homeostatic conditions and true inflammation named “para-inflammation” [6], which is an adaptive immunological response to low levels of “dangerous” stimuli such as environmental changes or mild air pollution that can lead to the progressive accumulation of oxidative stress over a period of months or years. The physiological role of para-inflammation is to maintain (or re-set) tissue homeostasis and restore its function [6]. Well-controlled para-inflammation may therefore be beneficial, whereas dysregulated para-inflammation is detrimental.

A number of studies have investigated how para-inflammatory responses become dysregulated in diseases affecting various tissues and organs, including the eyes [7,8,9,10], and there is some evidence that dysregulated para-inflammation is involved also in DED, which is a disease that affects the quality of life of more than 170 million people worldwide [11].

## 3. Dry Eye Disease as a Response to Tissue Stress

The various clinical forms of inflammation represent adaptive responses to tissue stress [12] that may occur when a number of cells suffer prolonged exposure to noxious changes in the micro-environment. However, the recent demonstration of a correlation between DED and laryngopharyngeal reflux suggests that the ocular surface may also be damaged by internal stresses. Although the exact mechanism of this process is not yet fully understood, it has been hypothesized that the toxic elements of gastric reflux may reach the ocular surface through the nasolacrimal ducts and give rise to epithelial damage and tear film instability [12].

## 4. Para-Inflammation as an Adaptive Response to Tissue Stress

Para-inflammation can occur when cells suffer repeated insults over a short period of time that are not strong enough to cause cell death (e.g., a change in microenvironmental parameters such as temperature, air movement, relative humidity, osmolarity, oxidative stress, etc.) [13]. At the cellular level, a low amount of stress induces an adaptive response that maintains cell viability but, when the stress becomes too intense, there is an inhibitory response characterized by the up-regulation of heat shock proteins and the activation of autophagic pathways [14].

Autophagy plays an important role in regulating the characteristics of ocular surface inflammation [15], but its protective effect against inflammation in DED decreases with age and anti-inflammatory therapy. Reduced autophagy can lead to the accumulation of dysfunctional organelles and thus further fuels the inflammatory response [16]. This is particularly relevant in the case of mitochondria because an increase in the number of dysfunctional mitochondria with reduced metabolic activity can increase the production of reactive oxygen species (ROS) and stimulate inflammatory responses by activating inflammasomes [17,18,19,20]. The autonomous response of cells (known as hormesis) is intended to allow a return to basal homeostasis, but this response may actually stimulate the production of pro-inflammatory cytokines and chemokines.

There is, therefore, a dual response regardless of the origin of the stressing stimuli: (a) a para-inflammatory tissue response involving resident macrophages/monocytes that may produce cytokines and growth factors in order to promote epithelial cell recovery, and (b) hormesis, which is initially an adaptive response intended to keep cells in good health but, when the stress is prolonged or particularly strong, subsequently induces the activation of apoptosis and consequent cell death.

If para-inflammation prevails, tissue repair can be achieved with minimal disturbance of the local immune system (“sub-clinical inflammation”); if the autonomous response of para-inflammation is insufficient in restoring the health of a large number of persistently stressed cells or a whole tissue, such as in the case of aging or during the course of DED, it can lead to cell malfunction or cell death.

Furthermore, unhealthy cells secrete pro-inflammatory cytokines and chemokines such as IL-1α/β, IL-6, IL-8, TNF-α, MCP-1/2, CX3CL1, insulin-like growth factor (IGF) and IGF receptors [21,22,23], which further stimulate resident macrophages and consequently initiate the adaptive immune pathway that makes the damage chronic [24].

Para-inflammation is regulated by the immune system of the tissue. This is mainly governed by resident macrophages and the complement system which, in turn, may also release cytokines and growth factors in order to promote the recovery of stressed cells [25,26].

Altered para-inflammatory mechanisms may be involved in the protraction and progression of DED. In young and healthy subjects, para-inflammation may maintain the homeostasis of the ocular surface, but prolonged stress and an age-related decline in autophagy may damage the ocular surface to the point of inducing a chronic disease in the presence of predisposing external risk factors, co-morbidities, high levels of oxidative stress, or pathogen/damage-associated molecular pattern (PAMP/DAMP) signaling [1,27]. In such cases, the failure to achieve system homeostasis leads to a persistent, sub-clinical and mainly asymptomatic inflammatory state that can fuel the onset or progression of chronic DED [25,26,28].

**Figure 1:**
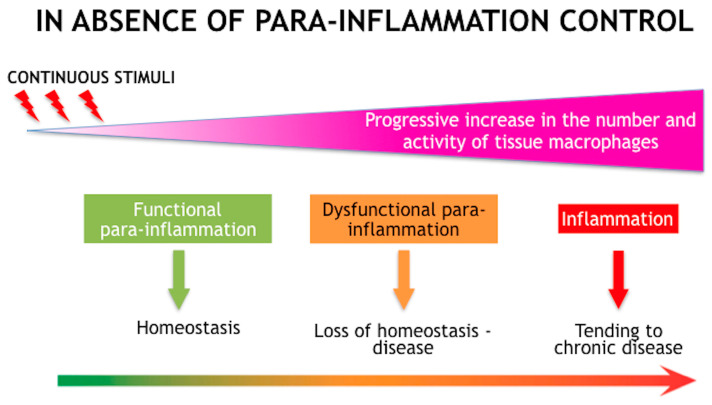
Schematic diagram showing the processes that lead from homeostasis of the ocular surface to chronic dry eye disease (DED). DED is characterized by internal and external continuous stimuli, which can induce stress of the ocular surface. In order to restore the system homeostasis, a hormetic response of adaptation and a para-inflammatory reaction is elicited. However, if the stimulus exceeds a threshold of time or intensity, homeostasis is lost and para-inflammation turns into inflammation. Adapted with permission from Ref. [11]. Copyright 2021 Taylor & Francis.

The simultaneous appearance of dysregulated homeostasis and chronic sub-clinical inflammation is the starting point for the development of a stable, chronic inflammatory disease that leads to clearly evident ocular surface damage (Figure 1) [11]. The oxidative process negatively affects not only the ocular surface but also the lipid layer of the tear film that, if oxidated, loses its fluidity and its ability to spread quickly over the mucosal-aqueous phase of the film, thus making it permeable to water and so eliminating its capacity to hinder tear film water evaporation.

The long-term follow-up of DED patients should consider all of the pathogenic issues involved in the disease, including the need to reduce the repeated or persistent external or internal stresses that are capable of activating and maintaining the para-inflammatory adaptive mechanisms of ocular surface structures, and which may lead to full-blown inflammation. It is particularly necessary to recognize such events quickly and to evaluate their persistence or any failure of the system to counteract the presence of inflammatory flare-ups [29]. It is essential to prevent, recognize and correct persistent sources of external stresses, such as excessive evaporation, in order to reduce their negative impact on the ocular surface. In addition to ensuring physical protection by means of lid hygiene and the appropriate preventive use of artificial tears, it is also important to control para-inflammation in order to avoid its dysregulation and correct it rapidly whenever a chronic inflammatory state is suspected as a result of the onset of discomfort symptoms.

Noxious environmental factors, which may also be associated with a patient’s lifestyle, can increase the risk of developing ocular surface damage, and changes in working conditions, exposure to air pollution, new topical or systemic therapies, or even a change of seasons can all require significant adaptation and possible therapeutic intervention [30].

Patients should be informed about the possibility of inflammatory flare-ups and the sudden worsening of the status of the ocular surface; in parallel, clinicians should be ready to take prompt action in order to resolve any problem as quickly as possible by identifying the possible causes and adapting previously prescribed treatments to the new conditions.

After a first diagnosis and treatment aimed at correcting alterations in ocular surface structures, long-term DED treatment is mainly preventive and needs to be adjusted on the basis of the condition of both the ocular surface and environmental factors. All ocular surface alterations induce DED and can be described in the form of a vicious circle consisting of various main stages: (1) tear film instability; (2) tear hyperosmolarity; (3) oxidative stress on tear film lipids and epithelial cells due to the inability of tear film to counteract it; (4) epithelial damage and the arrival of pro-inflammatory cytokines; (5) cell death due to apoptosis and inflammation; (6) nerve malfunction; and (7) anatomical and functional eyelid changes. Squamous metaplasia of the conjunctival epithelium and the loss of goblet cells also make a major contribution as they affect ocular surface lubrication and tolerance of resident saprophytic flora, in which any change increases the inflammatory stimulus [31]. The role of each of these factors in fueling the vicious circle needs to be recognized, evaluated, weighed and considered for appropriate treatment because only their correction can break the vicious circle and control the disease evolution [32].

Preventing exposure to persistent and possibly noxious stimuli should also be considered in order to assure permanent protection of the ocular surface and the prevention of DED.

## 5. Principles of Treatment

Protecting a subject from the exposome is an important aim of DED treatment, which also requires the identification of the main causative factors of the disease and the control of inflammation. Depending on the characteristics of the disease, this should be done by administering for a limited amount of time “smart and safe corticosteroids” that have a lower likelihood of causing adverse events [33,34] in order to restore the homeostasis of the ocular surface and then to protect the ocular surface from recurrent and potentially noxious stimuli like excessive water evaporation, tear hyperosmolarity and oxidative stress, which determine the induction of a detrimental para-inflammatory state.

Dysregulated para-inflammation can be corrected by means of the prolonged use of artificial tears containing minimal doses of safe steroids or other molecules in order to re-equilibrate ocular surface homeostasis. Although to date there are no published efficacy data, agents acting on the complement system like N-Acetylaspartylglutamic acid [35] or membrane stabilizers such as cromolyn [36] may be effective in reducing the secretion of a wide range of inflammatory mediators, including cytokines such as IL-1β, IL-6, IL-8 and IFN-γ and chemokines such as CXCL10, CCL2, CCL3 and CCL4, whose levels are typically high in DED [37]. Other tear substitutes can control excessive evaporation by adding polar lipids to the tear film lipid layer [38]. The correct and regular use of such tear substitutes can help to counteract the persistent pathogenic stresses on the ocular surface responsible for initiating and maintaining the vicious cycle of DED.

When available, mucin secretagogues represent an effective option for the treatment of DED [39]. They can be useful in cases of low BUT values because they are able to increase mucin production and therefore improve the interaction between the mucus and the aqueous part of the tear film. Mucins are glycoproteins expressed on the corneal and conjunctival cells surface that protect the cells and bind the water part of the tear film that contains numerous growth factors, enzymes and immunoglobulins. 

Topical cyclosporine has been used with success to treat DED with an inflammatory component, especially in cases of severe and chronic disease [40]. The target of cyclosporine is represented by T cells that are activated by antigen-presenting cells during the ocular surface inflammatory pathway. Its application is of fundamental importance for controlling inflammation in combination or as an alternative to topical corticosteroids. A European panel of experts recently agreed that in contrast to corticosteroids, cyclosporine can be continued indefinitely in DED patients, especially when surgery is required [41]. These strategies for controlling inflammation are important also in the setting of meibomian gland dysfunction since palpebral conjunctival epithelial and intraglandular inflammation has also been well documented in evaporative DED [42].

## Data Availability

No new data were created or analyzed in this study. Data sharing is not applicable to this article.
